# Estimating Risk of Circulatory Disease from Exposure to Low-Level Ionizing Radiation

**DOI:** 10.1289/ehp.1206046

**Published:** 2012-12-03

**Authors:** Helmut Schöllnberger, Jan Christian Kaiser

**Affiliations:** Helmholtz Zentrum München, Department of Radiation Sciences, Institute of Radiation Protection, Neuherberg, Germany, E-mail: schoellnberger@helmholtz-muenchen.de

The comprehensive meta-analysis of Little et al. (2012) summarized possible circulatory disease risks related to medium and low doses of whole-body radiation exposure in humans. The authors looked at excess relative risk (ERR) estimates from 10 different epidemiological studies. Using two statistical measures to calculate pooled ERR, they determined aggregate measures of ERR for four detrimental health outcomes and they reported mostly significant values for the ERR per unit dose in their Table 2.

Nine of the 10 studies Little et al. (2012) considered included moderate cumulative doses > 0.4 Sv (see their Table 1), and they observed that risk trends in most cohorts were driven by a relatively small number of highly exposed individuals. The authors then fitted a linear ERR model to the data of the meta-analysis and derived mortality risks at low-level radiation by extrapolation.

Linear extrapolation is used in radiation protection if cohort strata pertaining to low doses and dose rates have low statistical power. There are, however, indications for non-linear protective effects of low doses delivered at low dose rates for end points related to athero-sclerosis in mice (Mitchel et al. 2011). Moreover, the recent review of Rödel et al. (2012) showed that low-dose ionizing radiation modulates inflammatory immune reactions mostly with discontinuous or biphasic dose dependencies. These recent findings suggest that non-linear dose responses might also play a role in the determination of the radiation risk for circulatory diseases.

In this context we note that in the 10 studies analyzed by Little et al. (2012), risk estimates were mainly calculated with linear no-threshold (LNT) models (in fact, 7 of the 10 studies applied only the LNT model). Motivated by recent radio-biological findings, we fitted a large number of dose responses, in addition to the LNT model, to the data of the Life Span Study (LSS) cohort of Japanese atomic-bomb survivors, which is among the cohorts considered by Little et al. (2012). We realized that several models fitted the data about equally well (Schöllnberger et al. 2012). Instead of picking a single model of choice for risk assessment (here, the LNT model), we allowed for model uncertainty via multi-model inference. By reducing the bias from model selection, we obtained larger uncertainty intervals for risk estimates. The “model-averaged” dose response predicted markedly lower risks than the LNT model for cerebro-vascular disease (CVD) and for cardio-vascular diseases excluding CVD. For example, for CVD an ERR model with a step at 0.6 Sv strongly influenced the average with a weight of 0.55 compared with the LNT model with a much lower weight of 0.26 (see Table 1 of Schöllnberger et al. 2012). We did, however, not find any evidence for a protective effect but only for the contribution of pathways that have a threshold.

Our results might have implications for issues of public health in the assessment of risk–benefit ratios for radio-diagnosis or radio-therapy. Thus, we encourage the use of multi-model inference techniques in the analysis of other cohorts. From our experience with the LSS cohort, we would expect lower risk estimates in the lower dose range with a more comprehensive characterization of uncertainties and improved support of the epidemiological data.
